# The Analgesic Effect of Venlafaxine and Its Mechanism on Oxaliplatin-Induced Neuropathic Pain in Mice

**DOI:** 10.3390/ijms20071652

**Published:** 2019-04-03

**Authors:** Daxian Li, Ji Hwan Lee, Chang Won Choi, Jaihwan Kim, Sun Kwang Kim, Woojin Kim

**Affiliations:** 1 Department of Science in Korean Medicine, Graduate School, Kyung Hee University, Seoul 02447, Korea; lidaxian721@naver.com (D.L.); mibdna@khu.ac.kr (J.H.L.); 2 Department of East-West Medicine, Graduate School, Kyung Hee University, Seoul 02447, Korea; sistmein@naver.com (C.W.C.); boomjae@nate.com (J.K.); 3 Department of Physiology, College of Korean Medicine, Kyung Hee University, Seoul 02447, Korea

**Keywords:** chemotherapy-induced neuropathic pain, noradrenaline, oxaliplatin, serotonin, venlafaxine

## Abstract

The analgesic effect of venlafaxine (VLX), which is a selective serotonin and noradrenaline reuptake inhibitor (SNRI), has been observed on oxaliplatin-induced neuropathic pain in mice. Significant allodynia was shown after oxaliplatin treatment (6 mg/kg, i.p.); acetone and von Frey hair tests were used to assess cold and mechanical allodynia, respectively. Intraperitoneal administration of VLX at 40 and 60 mg/kg, but not 10 mg/kg, significantly alleviated these allodynia. Noradrenaline depletion by pretreatment of *N*-(2-Chloroethyl)-*N*-ethyl-2-bromobenzylamine (DSP-4, 50 mg/kg, i.p.) blocked the relieving effect of VLX (40 mg/kg, i.p.) on cold and mechanical allodynia. However, serotonin depletion by three consecutive pretreatments of para-chlorophenylalanine (PCPA, 150 mg/kg/day, i.p.) only blocked the effect of VLX on mechanical allodynia. In cold allodynia, the α_2_-adrenergic antagonist idazoxan (10 μg, i.t.), but not the α_1_-adrenergic antagonist prazosin (10 μg, i.t.), abolished VLX-induced analgesia. Furthermore, idazoxan and 5-HT_3_ receptor antagonist bemesetron (MDL-72222, 15 μg, i.t.), but not prazosin or mixed 5-HT_1, 2_ receptor antagonist methysergide (10 μg, i.t.), abolished VLX-induced analgesia in mechanical allodynia. In conclusion, 40 mg/kg of VLX treatment has a potent relieving effect against oxaliplatin-induced neuropathic pain, and α_2_-adrenergic receptor, and both α_2_-adrenergic and 5-HT_3_ receptors are involved in this effect of VLX on cold and mechanical allodynia, respectively.

## 1. Introduction

Oxaliplatin is a third-generation platinum-based chemotherapeutic agent widely used for different types of cancer [1,2]. It is reported to be safer than the first- and second-generation platinum-based agents, as it does not induce nephrotoxicity and hepatotoxicity [3]. However, oxaliplatin is known to induce peripheral neurotoxicity [1,4], which leads to the development of neuropathic pain. It is reported that about 90% of oxaliplatin-treated patients experience significant pain [5], as even a single injection can induce acute neuropathy within 24–48 h [6]. Cold and mechanical allodynia are common manifestations of this neuropathic pain [7]. To treat this oxaliplatin-induced pain, anticonvulsants and antidepressants, such as gabapentinoids and tricyclic antidepressants (TCAs) respectively, and serotonin and noradrenaline reuptake inhibitors (SNRIs) are generally used [8,9]. However, some reported that anticonvulsants and TCAs may be ineffective against oxaliplatin-induced neuropathic pain [10,11], and compared to SNRIs, anticonvulsants and TCAs are known to cause more adverse effects [11].

SNRIs enhance the action of serotonin and noradrenaline, which are both known to be involved in the endogenous analgesic mechanism. It is well known that enhancing the action of serotonin and noradrenaline can lead to pain attenuation [12,13]. Venlafaxine (VLX) is a SNRI, and studies reported that VLX is effective against neuropathic pain, such as painful diabetic neuropathy and poly-neuropathy in humans [14,15]. In addition, other clinical trials showed that VLX could be used to attenuate the neuropathic pain induced by chemotherapeutic agents [16,17]. However, compared to the large number of clinical studies, research conducted on animals is small. In addition, as clinical studies mostly focused on the analgesic effect of VLX [16,17,18], but rarely on its analgesic mechanism, the mechanism of VLX is still poorly understood [17].

Over the past several years, we found an effective treatment method to attenuate oxaliplatin-induced neuropathic pain [3,19,20,21]. Various types of different treatment methods were used, and our data showed that the noradrenergic and serotonergic systems are important in relieving the pain caused by oxaliplatin. Activating the spinal adrenergic receptors via its agonist significantly reduced the upregulated spinal neuronal cells after oxaliplatin injection [22], and also blocking the adrenergic and serotonergic receptors by their antagonists greatly reduced the analgesic effect of the treatments [22,23,24,25,26].

Thus, in this study we focused on the effect of VLX, a noradrenaline and serotonin transporter inhibitor, against oxaliplatin-induced neuropathic pain. We firstly observed whether three different doses of VLX could significantly attenuate the neuropathic pain and the elapsed time of their effect was also investigated. Secondly, we clarified the role of noradrenaline and serotonin by systematically depleting them. Finally, by injecting adrenergic and serotonergic receptor antagonists intrathecally, we investigated which receptor subtypes present at the spinal cord play a major role in the effect of VLX.

## 2. Results

### 2.1. Time Course of the Anti-Allodynic Effects of Venlafaxine (VLX) in a Mouse Model of Oxaliplatin-Induced Cold and Mechanical Allodynia

**Figure 1:**
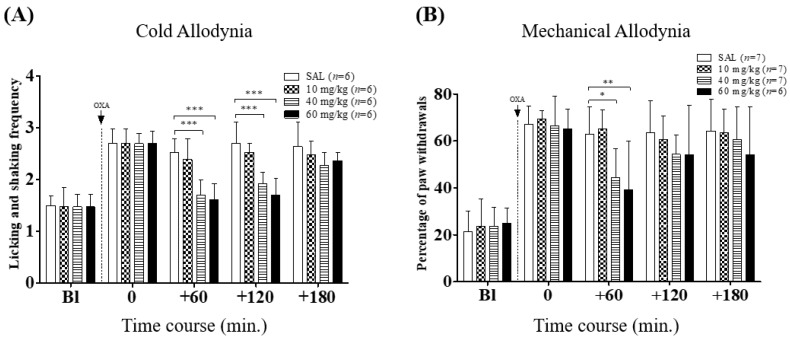
Elapsed time of the anti-allodynic effects of venlafaxine (VLX) on oxaliplatin-induced cold (**A**) and mechanical (**B**) allodynia in mice. Mice were divided arbitrarily into four groups. ‘SAL’ refers to saline, which was used as control, and “10”, “40” and “60 mg/kg” refers to the doses of VLX used. On the timeline, “Bl” refers to the baseline measured prior to the administration of oxaliplatin (OXA, 6 mg/kg, i.p., dotted line). “0” refers to the measurements performed just before the treatment of VLX or saline on day four. Data was expressed as mean ± standard deviations (S.D.). * *p* < 0.05, ** *p* < 0.01, *** *p* < 0.001, vs. saline with two-way ANOVA followed by Bonferroni’s post-test.

First, we evaluated the analgesic effects of VLX on oxaliplatin-induced cold and mechanical allodynia. VLX at three different doses (10, 40 and 60 mg/kg, i.p.) were administered four days after the single oxaliplatin injection (6 mg/kg, i.p.), when both the cold and mechanical allodynia were significantly shown. As compared to the control (saline; SAL group), both 40 and 60 mg/kg of VLX exerted significant suppressive effects, lasting for 120 and 60 min, on cold and mechanical allodynia, respectively (Figure 1). The highest dose of VLX (60 mg/kg) was shown to have slightly stronger analgesic effects than the intermediate dose (40 mg/kg), but neither of the effects of these two doses lasted up to 180 min after the drug injections. The lowest dose of VLX (10 mg/kg) failed to show significant relieving effects compared to the control at any time point on cold and mechanical allodynia (Figure 1). Based on these results, 40 mg/kg of VLX was used in the following experiments.

### 2.2. Development of Cold and Mechanical Allodynia by a Single Intraperitoneal Administration of Oxaliplatin in Noradrenaline or Serotonin Depleted Mice

**Figure 2:**
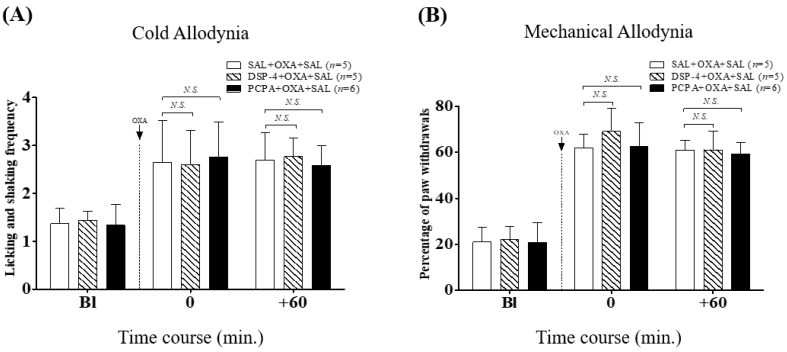
Effects of noradrenaline or serotonin depletion on cold (**A**) and mechanical (**B**) allodynia induced by oxaliplatin administration. Before oxaliplatin (OXA, 6 mg/kg, i.p., dotted line) administration, mice were treated with single DSP-4 (50 mg/kg, i.p.) or three consecutive para-chlorophenylalanine (PCPA) injections (150 mg/kg/day, i.p.). On day four, the measurements were performed before the injection of saline (SAL) and 60 min after. “Bl”, baseline; *N.S.*, no significance (*p* > 0.05), vs. SAL + OXA + SAL. Data was expressed as mean ± S.D. and analyzed with two-way ANOVA followed by Bonferroni’s post-test.

To observe whether *N*-(2-Chloroethyl)-*N*-ethyl-2-bromobenzylamine hydrochloride (DSP-4) and para-chlorophenylalanine (PCPA) affect the development of oxaliplatin-induced neuropathic pain, mice were treated with single DSP-4 (50 mg/kg, i.p.) or multiple PCPA (150 mg/kg/day, for three days, i.p.) to deplete noradrenaline and serotonin, respectively. Results showed that neither DSP-4 nor PCPA pretreatment exerted an influence on the severity of cold and mechanical allodynia, as no significant differences were shown compared to the control (SAL + OXA + SAL group, Figure 2). These results indicate that both noradrenaline and serotonin have limited contribution to the development of cold and mechanical allodynia induced by a single treatment (6 mg/kg, i.p.) of oxaliplatin.

### 2.3. Effects of VLX on Oxaliplatin-Induced Cold and Mechanical Allodynia in Noradrenaline-Depleted Mice

**Figure 3:**
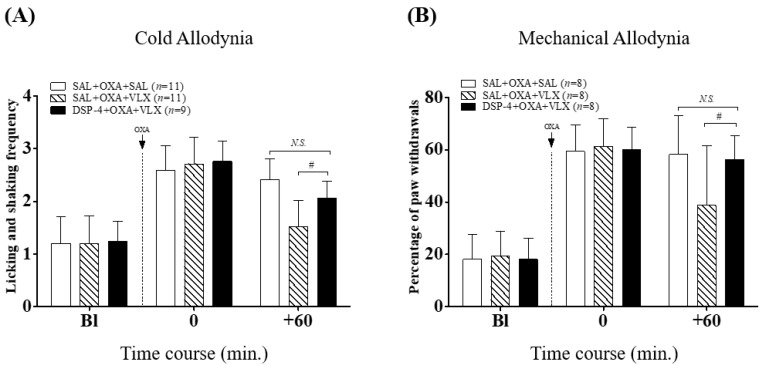
Roles of noradrenaline on the relieving effects of VLX on oxaliplatin-induced cold (**A**) and mechanical (**B**) allodynia in mice. Oxaliplatin (OXA, 6 mg/kg, i.p., dotted line) was injected one day after the DSP-4 (50 mg/kg, i.p.) or saline (SAL, vehicle) treatment. On day four, mice were subjected to the measurements just before the VLX (VLX, 40mg/kg, i.p.) or saline (SAL) injection and 60 min after the treatments. “Bl”, baseline; ^#^ *p* < 0.05, vs. SAL + OXA + VLX; *N.S.*, no significance (*p* > 0.05), vs. SAL + OXA + SAL. Data was expressed as mean ± S.D. and analyzed with two-way ANOVA followed by Bonferroni’s post-test.

Next, we investigated the role of noradrenergic system in the anti-allodynic effect of VLX by conducting behavioral tests 60 min after its administration on day four, when significant allodynia signs were shown. Compared to control (SAL + OXA + SAL group), VLX (40 mg/kg, i.p., SAL + OXA + VLX group) administration significantly attenuated cold and mechanical allodynia (Figure 3, *p* > 0.05). However, pretreatment of DSP-4 significantly reversed this analgesic effect of VLX in both kinds of allodynia (DSP-4 + OXA + VLX group vs. SAL + OXA + VLX group, *p* < 0.05, respectively). These results reveal that the noradrenergic system is involved in VLX-induced analgesic actions on cold and mechanical allodynia in oxaliplatin-administered mice.

### 2.4. Roles of Serotonergic Pathway on the Anti-Allodynic Effects of VLX in Oxaliplatin-Administered Mice

**Figure 4:**
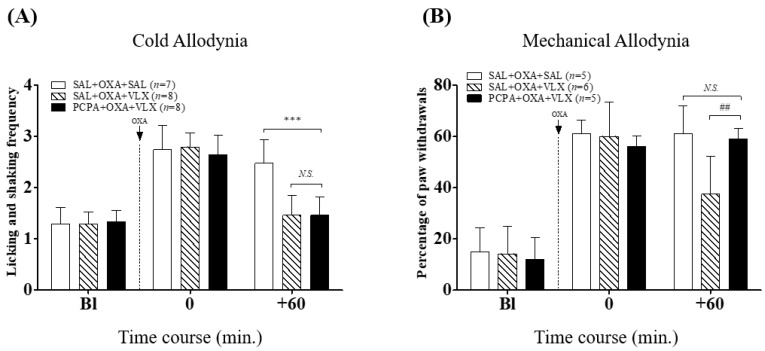
Roles of serotonergic pathway on the relieving effects of VLX on oxaliplatin-induced cold (**A**) and mechanical (**B**) allodynia in mice. PCPA (150 mg/kg/day, i.p.) or saline (SAL, vehicle) was administered before the oxaliplatin (OXA, 6 mg/kg, i.p., dotted line) treatment. On day four, mice underwent the measurements twice; just prior to the VLX (VLX, 40mg/kg, i.p.) or saline (SAL, vehicle) injection, and 60 min after the treatments. “Bl”, baseline; *** *p* < 0.001, vs. SAL + OXA + SAL; ^##^ *p* < 0.01, vs. SAL + OXA + VLX; *N.S.*, no significance (*p* > 0.05). Data was expressed as mean ± S.D. and analyzed with two-way ANOVA followed by Bonferroni’s post-test.

In our subsequent studies, we examined the role of serotonin (5-HT) on the analgesic effect of VLX by depleting the serotonin through PCPA administration. In the acetone test (Figure 4A) 40 mg/kg of VLX significantly reduced the hind paw frequency of licking and shaking following acetone stimuli, and PCPA pretreatment did not prevent this effect (SAL + OXA + VLX vs. SAL + OXA + SAL, PCPA + OXA + VLX). However, on mechanical allodynia, PCPA pre-administrations entirely blocked the analgesic effect of VLX, as it completely blocked the effect of VLX (vs. SAL + OXA + VLX group, *p* < 0.01). These results demonstrate that the serotonergic pathway is involved in the VLX-induced analgesic action on mechanical allodynia, but not on cold allodynia in oxaliplatin-administered mice.

### 2.5. Spinal Mechanisms of α-Adrenergic Receptors on VLX-Induced Analgesia in Cold Allodynia

**Figure 5:**
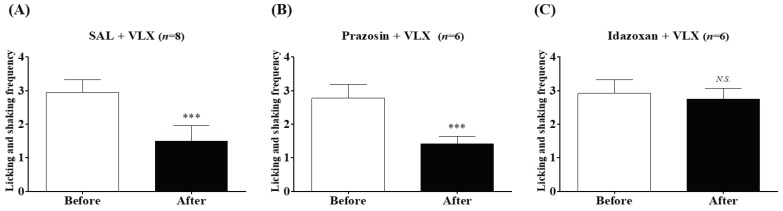
Effects of intrathecal injection with α-adrenergic antagonists on the analgesic action of VLX against cold allodynia. Mice with cold nociception were divided arbitrarily into three groups: saline (SAL), prazosin (10 μg) and idazoxan (10 μg) were intrathecally (i.t.) injected 20 min before VLX (VLX, 40mg/kg, i.p.) treatment (**A**–**C**, respectively). Measurements were performed twice; just before the intrathecal injection (Before) and 60 min after VLX treatment (After). Data was expressed as mean ± S.D.; *N.S.*, no significance (*p* > 0.05); *** *p* < 0.001, vs. Before; by paired *t*-test.

To further demonstrate which α-adrenergic receptor subtypes mediate the VLX-induced analgesic action against cold allodynia at the spinal level, prazosin (α_1_-adrenergic antagonist) or idazoxan (α_2_-adrenergic antagonist) was injected intrathecally in a volume of 5 μL, 20 min before the VLX (40 mg/kg, i.p.) administration. Figure 5 shows that idazoxan (10 μg), but not prazosin (10 μg) or vehicle (SAL) reversed the relieving effects of VLX, revealing that VLX alleviates cold allodynia through the activation of spinal α_2_-adrenergic receptors, but not α_1_-adrenergic receptors.

### 2.6. Spinal Mechanisms of α-Adrenergic or Serotonergic Receptor on the VLX-Induced Analgesia in Mechanical Allodynia

**Figure 6:**
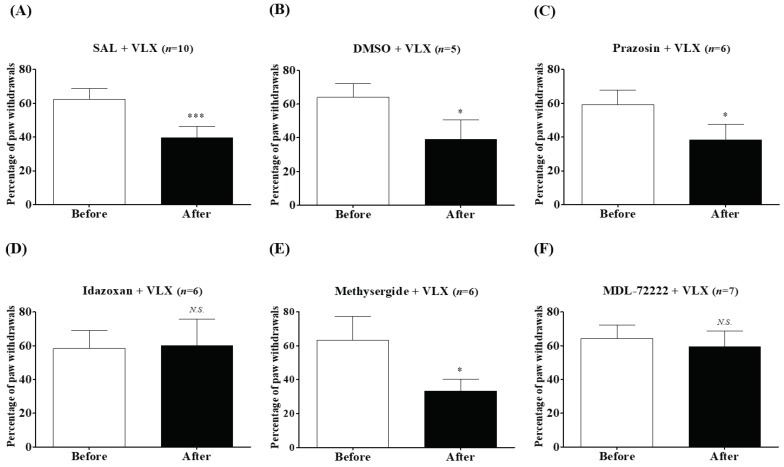
Effects of intrathecal injection with α-adrenergic or serotonergic antagonists on the analgesic action of VLX against mechanical allodynia. Mice with mechanical nociception were divided arbitrarily into six groups: saline (SAL), DMSO, prazosin, idazoxan, methysergide and bemesetron (MDL-72222) were intrathecally (i.t.) injected 20 min before VLX (VLX, 40mg/kg, i.p.) treatment (**A**–**F**, respectively). Then measurements were performed just before the i.t. injection (Before) and 60 min after VLX treatment (After). Data was expressed as mean ± S.D.; *N.S.*, no significance (*p* > 0.05); * *p* < 0.05, *** *p* < 0.001, vs. Before; by paired *t*-test.

Finally, to identify which α-adrenergic or serotonergic receptor subtypes mediate the VLX-induced analgesic action against mechanical allodynia at the spinal level, prazosin, idazoxan, methysergide (mixed 5-HT_1, 2_ receptor antagonist), or bemesetron (MDL-72222,5-HT_3_ receptor antagonist) was injected intrathecally in a volume of 5 μL, 20 min before the treatment. As shown in Figure 6, prazosin (10 μg) and methysergide (10 μg), along with two other vehicles (SAL and DMSO), induced a significant decrease in the percentage of paw withdrawals after VLX treatments. In contrast, in the idazoxan (10 μg) and MDL-72222 (15 μg) pretreated mice, the VLX-induced analgesia was mainly blocked (Figure 6D,E). Thus, these results show that activation of α_2_-adrenergic and 5-HT_3_ receptors, but not α_1_-adrenergic or 5-HT_1, 2_ receptors, contribute to the relieving actions of VLX on mechanical allodynia.

## 3. Discussion

In this study, we investigated the analgesic effect of VLX on oxaliplatin-induced neuropathic pain, and clarified the mechanism of its effect. Three different doses of VLX were used; 10, 40, and 60 mg/kg. However, 40 and 60 but not 10 mg/kg of VLX were shown to attenuate the pain induced by oxaliplatin injection. The effects of both 40 and 60 mg/kg of VLX lasted until 120 min after the administration on cold allodynia, whereas on mechanical allodynia, their effects were not observable at 120 min (Figure 1). This data shows that VLX may be more effective against cold than mechanical allodynia. Similarly, the other group also reported that VLX only reversed the cold but not mechanical allodynia induced by oxaliplatin, suggesting that VLX may be more effective against cold than mechanical allodynia [27]. In our subsequent experiments, we used 40 mg/kg of VLX, as 10 mg/kg was not potent enough against oxaliplatin-induced pain, and also as the effect of 60 mg/kg of VLX was not significantly different from that of the 40 mg/kg. VLX was reported to be more effective when used at a dose above 30 mg/kg as it acts as a serotonin and noradrenaline transporter inhibitor, whereas at a dose below 10 mg/kg, it only acts as a serotonin reuptake inhibitor [28]. This may explain why in our experiment, the lowest dose of VLX (10 mg/kg) was not effective against oxaliplatin-induced neuropathic pain.

In the next experiments, we used DSP-4 and PCPA to elucidate the underlying mechanisms of VLX, by depleting endogenous noradrenaline and serotonin, respectively. DSP-4 and PCPA injection did not cause any effect on the development of cold and mechanical allodynia (Figure 2); however, single pretreatment of DSP-4 blocked the analgesic effect of VLX on both cold and mechanical allodynia (Figure 3). PCPA pretreatment only blocked the effect of VLX on mechanical allodynia, showing that the serotonergic system is not involved in the analgesic effect of VLX against cold allodynia induced by oxaliplatin (Figure 4).

For the next step, we focused on the action of adrenergic and serotonergic receptors subtypes present at the spinal cord, as noradrenalin and serotonin contents in the spinal cord were shown to be up-regulated after VLX treatment [29,30]. Our results showed that α_2_-adrenergic, but not α_1_-adrenergic receptors are involved in the action of VLX against cold allodynia (Figure 5), whereas on mechanical allodynia, both α_2_-adrenergic and 5-HT_3_ receptors were shown to be involved (Figure 6). Spinal α_2_-adrenergic receptors are known to play an important role in the analgesic effect, as activating spinal α_2_-noradrenergic receptors by using its agonist clonidine resulted in pain attenuation in various animal models of pain [31,32]. Moreover, in our previous studies conducted on oxaliplatin-induced neuropathic pain, blocking the spinal α_2_-noradrenergic receptors by its antagonists blocked the analgesic effect of other treatments [22,24,25,26]. However, in another study [33], we reported that duloxetine, a serotonin-noradrenaline reuptake inhibitor similar to VLX, could also decrease oxaliplatin-induced cold and mechanical allodynia, by acting on α_1_-adrenergic receptors, as the effect of duloxetine was blocked by an α_1_-(prazosin)_,_ but not α_2_-(idazoxan) adrenergic receptor antagonist. Altogether, these results suggest that activation of both α_1_- and α_2_-adrenergic receptors may suppress the allodynia induced by oxaliplatin [22].

Serotonin, along with noradrenaline, is a critical neurotransmitter deeply involved in the endogenous pain inhibitory system [13], and 5-HT_3_ receptors are known to be involved in pain attenuation as they are reported to be largely present on the inhibitory interneurons of the spinal dorsal horn [34]. In our study, the serotonergic system was not involved in the analgesic effect of VLX against cold allodynia induced by oxaliplatin. Although it is difficult to clarify the reason for this difference with our own data, the mechanism of cold and mechanical allodynia has been reported to be different. The cold allodynia is mostly mediated by unmyelinated C fibers [35,36,37], whereas mechanical allodynia is mediated by both A and C fibers [38]. A fibers are known to innervate both in the superficial and deep laminae of the spinal dorsal horn, whereas C fibers mostly innervate in the superficial layer of the dorsal horn. On the superficial layer, inhibitory interneurons are widely present and death of these interneurons were reported to contribute to pain [39,40]. Moreover, their activities can be modulated by serotonin as they possess serotonin receptors [41,42]. However, as it was reported that the number of interneurons could decrease following a nerve damage [43], we suppose that oxaliplatin injection may have lessened the role of these interneurons, decreasing the analgesic action of serotonin on C fibers, but more detailed experiments are needed to clarify this.

In conclusion, our results show that 40 mg/kg of VLX treatment has a significant analgesic effect against oxaliplatin-induced neuropathic pain. Noradrenaline was shown to mediate its effect on both cold and mechanical allodynia, whereas serotonin was only involved in the action against mechanical allodynia. Furthermore, VLX was shown to alleviate cold allodynia through the activation of spinal α_2_-adrenergic receptors, but not α_1_-adrenergic receptors. On mechanical allodynia, activation of α_2_-adrenergic and 5-HT_3_ receptors, but not α_1_-adrenergic or 5-HT_1, 2_ receptors, were shown to contribute to the relieving actions of VLX.

## 4. Materials and Methods

### 4.1. Animals

Young adult, male C57BL/6 mice (six weeks old) weighing 18 to 25 g were obtained from Daehan Biolink (Chungbuk, Korea). They were housed four per cage under controlled temperature (23 ± 2 °C) and humidity (65% ± 5%) with food and water available ad libitum under standard pathogen-free laboratory conditions. Artificial lighting was maintained on a fixed 12 h light/dark cycle (a light cycle; 07:00–19:00, a dark cycle; 19:00–07:00). Animals were habituated to the behavioral testing environment with handling procedures by the investigator at least one week before the beginning of experiments. All experimental protocols were approved by the Kyung Hee University Animal Care and Use Committee (KHUASP (SE)-16-153, 29 December 2016).

### 4.2. Oxaliplatin or VLX Administration

Oxaliplatin (Sigma, St. Louis, MO, USA) was dissolved in a 5% glucose solution at a concentration of 2 mg/mL and was intraperitoneally (i.p.) injected at 6 mg/kg bodyweight [44,45]. As control, the same volume of 5% glucose solution was intraperitoneally injected.

VLX (Sigma) was dissolved in saline (SAL) at three different concentrations (1, 4 and 6 mg/mL) and was intraperitoneally injected at doses of 10, 40 and 60 mg/kg, respectively [46,47]. Also, the control group received the same volume of saline (SAL).

### 4.3. Behavioral Tests

The first aim of this study was to investigate the dose-dependent anti-allodynic effects of VLX in a mouse model of oxaliplatin-induced hypersensitivity. In our previously published works [24,25], the significant cold and mechanical allodynia symptoms were ascertained from three days after a single oxaliplatin (6 mg/kg, i.p.) injection and maintained up to nearly seven days. Therefore, in this study, behavioral tests were performed before the administration of oxaliplatin (Bl), prior to the treatment of VLX in day four (time point 0), and resumed 60, 120 and 180 min after the injections of VLX (+60, +120, +180, respectively). The investigator was blinded to the usages of all drugs. Behavioral tests were only performed during the light period.

To quantify cold allodynia, brisk reactions of hind paw in response to acetone stimuli were measured [48,49]. Mice were caged beneath an inverted clear plastic cage (12 × 8 × 6 cm) atop a metal mesh floor and enabled to acclimate for 30 min prior to the measuring. Acetone (10 μL, Reagents Chemical Ltd., Gyonggi-do, Korea) was applied to the mid-plantar skin of each side three times and the frequencies of brisk withdrawal, licking and shaking of the testing hind paws were counted after the acetone stimuli for 30 s.

To quantify mechanical allodynia, quick hind paw withdrawal induced by von Frey filament application was measured [48,50]. Mice were caged beneath an inverted clear plastic cage (12 × 8 × 6 cm) atop a metal mesh floor and enabled to acclimate for 30 min before the measuring. A von Frey hair (Linton Instrumentation, Norfolk, UK) with a bending force of 0.4 g was applied to the mid-plantar skin of each side 10 times (once every 5 s). The number of brisk withdrawal reactions to the von Frey filament stimuli from both hind paws was counted and expressed as an overall percentage response.

### 4.4. Depletion of Noradrenaline or Serotonin

To estimate whether and how the noradrenergic or serotonergic system mediates the anti-allodynic mechanism of VLX in oxaliplatin-administered animals, two different groups of mice were treated with *N*-(2-Chloroethyl)-*N*-ethyl-2-bromobenzylamine hydrochloride (DSP-4) or Para-chlorophenylalanine (PCPA), respectively. The formulae of DSP-4 and PCPA were determined according to the previous works [51,52,53,54], which could widely deplete correlative neurotransmitter stores in the central nervous system level (noradrenaline and 5-HT, respectively).

Briefly, animals were intraperitoneally injected with DSP-4 (Tocris, Cookson, UK, 50 mg/kg, 5 mg/mL) or vehicle (SAL) a day before the oxaliplatin administration [51,52]. On the other hand, PCPA (Sigma, 150 mg/kg/day, 15 mg/mL) or vehicle (SAL) was intraperitoneally injected to animals prior to oxaliplatin treatment for three days [53,54].

### 4.5. Adrenergic or Serotonergic Receptor Antagonist Administration

To further examine which adrenergic or serotonergic receptor subtype at the spinal cord level mediates the analgesic effects of VLX in oxaliplatin-administered mice, four specific antagonists were administered intrathecally (i.t.) 20 min prior to the VLX administration. The formulae of antagonists were determined based on previous reports [23,25,55]. Concisely, α_1_-adrenergic receptor antagonist prazosin (10 μg, Sigma), α_2_-adrenergic receptor antagonist idazoxan (10 μg, Sigma), a mixed 5-HT_1, 2_ receptor antagonist methysergide maleate (10 μg, Tocris, Cookson, UK) were dissolved in saline. Besides, 5-HT_3_ receptor antagonist MDL-72222 (15 μg, Tocris) was dissolved in 20% DMSO [23,56]. All antagonists or vehicles were administered intrathecally in volumes of 5 μL. In a prone position, mice were anaesthetized with a combination of 2–2.5% isofluorane in N_2_O/O_2_ (50:50 *v*/*v*), and then a Hamilton syringe needle was inserted into the subarachnoid space in L5–L6 intervertebral level [57,58].

### 4.6. Statistical Analysis

Data were presented as mean ± S.D. Statistical analysis was done with the software of Prism 5.0 (Graph Pad Software, San Diego, CA, USA, 2008). Paired *t*-test or two-way ANOVA followed by Bonferroni’s multiple comparison test was used for statistical analysis. In all cases, *p* < 0.05 was considered statistically significant.
